# Lymphoepithelioma-like carcinoma of the breast: a case report and review of the literature

**DOI:** 10.11604/pamj.2019.32.18.15978

**Published:** 2019-01-11

**Authors:** Kouhen Fadila, Abbad Faycal, Jabri Lamiaa, Belghiti Mohammed, Ismaili Nabil

**Affiliations:** 1 Department of Radiotherapy, International University Hospital Sheikh Khalifa, Mohammed VI University of Health Sciences (UM6SS), Casablanca, Morocco; 2 Department of Pathology, International University Hospital Sheikh Khalifa, Mohammed VI University of Health Sciences (UM6SS), Casablanca, Morocco; 3 Department of Pathology, Specialty Hospital, Rabat, Morocco; 4 Department of Medical Oncology, International University Hospital Sheikh Khalifa, Mohammed VI University of Health Sciences (UM6SS), Casablanca, Morocco

**Keywords:** Breast cancer, lymphoepithelioma-like carcinoma, rare

## Abstract

Lymphoepithelioma-like carcinoma of the breast is uncommon with only 21 patients documented in the literature. It can wrongly be diagnosed as medullary carcinoma and certain types of lymphoma due to undifferentiated proliferation of malignant epithelial cells with prominent lymphoid infiltration. In this paper, we present a case of LELC of the breast in a 64-year-old female with breast LELC and a discussion based on a review of the literature.

## Introduction

Lymphoepithelioma carcinoma is a rare entity. It was first identified in the nasopharynx in 1921 by Regaud and Schminke. More rarely, LELC sits on the lung, esophagus, stomach, skin, cervix, kidney and the breast [1]. Lymphoepithelioma-like carcinoma of the breast is extremely rare [2]. The first case was being reported by Kumar and Kumar [3], then, only 22 cases have been reported in the literature [4]. It is an undifferentiated carcinoma composed of malignant epithelial cells with prominent lymphoid infiltration and can wrongly be diagnosed as medullary carcinoma and certain types of lymphoma. This paper adds one more case to the published literature. We present a case of LELC of the breast in a 64-year-old female and a discussion based on a review of the literature.

## Patient and observation

**Figure 1:**
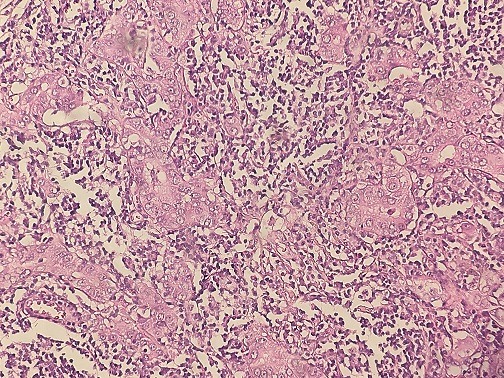
Invasive carcinomatous component with lymphoid stroma x20

**Figure 2:**
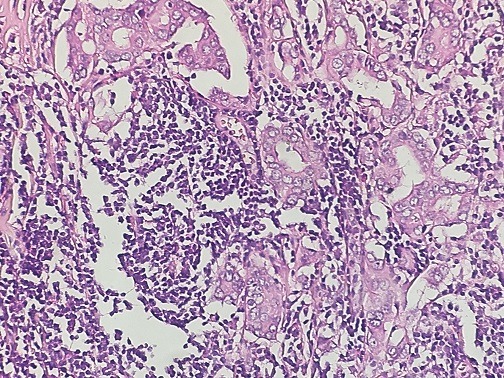
Stromal component made of mononuclear cell (lymphocytes and plasma cells x20

A 64-year-old woman was admitted to our institution with palpable lump in her left breast. Patient had not any previous medical and family story of cancer and denied any use of alcohol and cigarettes. She took oral contraception for 5 years. Her menarche was at age 12. Physical examination revealed a 3cm tumor located on the left breast adhere to deep plans. There was no retraction of the nipple, skin ulceration or inflammatory changes. The right breast exam was negative and there was no clinical evidence of axillary lymph node involvement. Mammography revealed a 2.6cm round hyperdense mass with irregular and speculated margins in the subareolar area with associated microcalcifications within the mass. The mass was categorized as Breast Imaging Reporting and Data System category 5. Fine needle aspiration and a core biopsy of the lesion were performed and the diagnostic was tubular carcinoma of the breast. The patient underwent left lumpectomy with axillary node dissection. The macroscopic (gross) examination of specimens revealed the presence of a nodule measuring 1.5cm in its largest diameter. The histology showed that tumor cells were arranged in a rare trabecular pattern with a prominent lymphoid stroma. Carcinomatous cells were polygonal with granular amphophilic cytoplasm and a nucleus with fine chromatin. Nucleoli were generally inconspicuous. Up to 8 mitoses per 10 HPF were counted. No lympho-vascular component or intraductal component was noted (Figure 1, Figure 2). The tumor was grade II of Scarf Bloom Richardson. Surgical margin was clear. The resected axillary lymph nodes contained metastases, including one/19 N level I lymph nodes. The immunohistochemistry study showed an expression of the following antibodies Ck (AE1/AE3) on the carcinomatous component, CD3 and CD20 with homogenous distribution in the stroma. The Ki-67 labeling index was: 40% (estimated on 10HPF), Estrogen receptor status were 90%, progesterone receptor 10% (both using Allred score) and the human epidermal growth factor was negative. Based on the histopathological and immunohistochemical findings the case has been reported as lymphoepithelioma-like carcinoma of the breast and staged as pT1N1M0. The post-operative pet scan showed no distant metastasis. The laboratory data showed a normal level of carbohydrate antigen 15-3(CA15-3: 39.8 U/ml). Following surgery, the patient underwent four cycles of doxorubicin and cyclophosphamide and twelve cycles of paclitaxel chemotherapy. She declined endocrine therapy and was treated with radiotherapy commencing 3 weeks after chemotherapy. The radiation dose was 50Gy in 25 fractions using a 6MV photon tangent pair followed by a boost of 10Gy in 5 fractions using 6MV photon tangent pair. Regular follow up consists of physical examination every three months. At her 1 year follow-up, the patient was doing well with no evidence of recurrent disease.

## Discussion

Lymphoepithelioma carcinoma (LELC) is an undifferentiated carcinoma with an intermixed reactive lymphoplasmatic infiltrate [5]. To our knowledge, 23 cases of LELC of the breast, including the present case, have been reported since the first Report in 1994 by Kumar and Kumar. Epstein-Barr virus (EBV) and human papilloma virus (HPV) have been cited for their possible association with LELC of the breast [1, 6], not tested in our patient. The differential diagnosis of lymphoepithelioma-like carcinoma (LELC) includes medullary carcinoma and certain types of lymphoma [7, 8]. The clinical presentations and the radiology findings of LELC are similar to that of other primary breast cancer. Nodal involvement was reported in only five cases and no distant metastases were found in in the 22 cases reported in the literature. Nine cases were positive for the hormone receptors. HER2 was positive in 3 of 16 patients who had this staining. Histologically, it is an undifferentiated carcinoma with an intermixed reactive lymphoplasmacytic infiltrate. Although there is some histologic overlap between atypical medullary carcinoma and the Regaud type of lympho-epithelioma-like carcinoma, the absence of syncytial sheets of tumor cells in the current case favors the latter diagnosis. In their description of lymphoepithelioma-like carcinoma of the breast, Kumar and Kumar22 did not mention the distinction of this tumor from atypical medullary carcinoma [9]. The use of immunohistochemistry is helpful in distinguishing of LELC lesions, which differ in terms of prognosis and treatment. In LELC, tumor cells always express cytokeratin and EMA. The lymphoid cells of the stroma-reaction are in the majority of the cases of phenotype T: CD3 +, CD8 + mixed with some B lymphocytes. Owing to the rarity of this special histological type of breast cancer, there are no clear guidelines to define optimal oncologic management. All cases reported in the literature have been treated surgically: seven mastectomies, six lumpectomies, seven wide local excisions, and in one case surgery was not reported. Adjuvant radiotherapy was delivered to eight patients. The role of systemic chemotherapy is not clear. Only 7 patients (32%) underwent chemotherapy. Our case underwent wide locale excision, four cycles of doxorubicin and cyclophosphamide, twelve cycles of paclitaxel adjuvant chemotherapy and adjuvant radiotherapy with a radiation dose of 50Gy in 25 fractions followed by a boost of 10Gy in 5 fractions. Lymphoepithelioma-like carcinoma has an often favorable outcome [10]. Our patient remained disease free at the time of her last follow-up examination (20 months after primary surgery).

## Conclusion

In summary, LELC of the breast is a rare diagnosis with distinct morphological features and a usually good prognosis. Its pathogenesis remains unclear and seems to be multifactorial. To the best of our knowledge, this is the 23 report of this rare entity occurring in 64 years old female with disease free of 20 months.

## Competing interests

The authors declare no competing interests.
